# Supplementary material for: Chromothripsis during telomere crisis is independent of NHEJ, and consistent with a replicative origin
Source: Genome Res. 2019 May;29(5):737–49. doi: 10.1101/gr.240705.118 (PMC6499312; doi:10.1101/gr.240705.118)
Supplement: Supplemental Material [file supp_gr.240705.118_Supplemental_file_1.zip › contigs/annotated_contigs/DB50/contig.2.DB50_length_568_mean_cov_8.25176056338.docx]

**DB50_length_568_mean_cov_8.25176056338**

```
GTCACTGCTGATAGTATGATAC|TTGGCACAACCACTTTGGGAAACCATGTGGAAGAATTCTTAACAGCTCAACAAATCCACATCTTAT
                       >chr22:48521103-48521314 + E=3e-112
GCACAGCAATTTCATTTCTAGGTTCACGTCTCACAGAAACGCATATGTGAGTTCTTCTAAAGACATGAAAAGTTTATAAAGGCACTATT

CATTACCACATAACTAGGAATTTCCCAAACGCCCATCAATAATGGAACAGACACAT|AAATTAGCCAGGCATGGTGGTGCATGCCTGTA
                                                          >chr22:48524029-48524365 + E=9
ATCCCAAATACTTGGGAGGCTGAGGCAGGAGAATCACTTGAACCCAGGAGGCAGAGGTTGCGGCGAGCTGAGATTGCGCCATTGCACTC
e-186
CAGCTTGGGTGACAAAGTGTGTCTCTGTCTAAAAATAAAAAAAAAAAGAATCAACTCATATAAAGAATGTATACAAGAAGTCCTTTATC

ACAATAGTAAAAGCAAAACAGCCCAACATAAAAGTGACCCAAATACATGAGCAGTTATTGTACACAAAAGGAAAAACGTAGAGGCTGTA

GACAGATGAAGAGATGTTCAGCCTCATTAGTCAATA
```
